# Successful Endovascular Repair of an Abdominal Aortic Aneurysm in a Patient with a Horseshoe Kidney and Accessory Renal Arteries Using an Aortic Cuff to Prevent Type II Endoleak

**DOI:** 10.3400/avd.cr.20-00099

**Published:** 2021-03-25

**Authors:** Takuma Mikami, Takeshi Kamada, Hiroki Uchiyama, Yosuke Kuroda, Ryo Harada, Syuichi Naraoka, Nobuyoshi Kawaharada

**Affiliations:** 1 Department of Cardiovascular Surgery, Sapporo Medical University School of Medicine, Sapporo, Hokkaido, Japan

**Keywords:** abdominal aortic aneurysm, endovascular aneurysm repair, horseshoe kidney

## Abstract

A 78-year-old man presented with an abdominal aortic aneurysm (AAA) and a horseshoe kidney coexisting with accessory renal arteries. We performed surgical treatment with endovascular aneurysm repair, sacrificing the accessory renal arteries. We used an aortic cuff to prevent a type II endoleak from the inferior mesenteric and accessory renal arteries. Decreased renal function was transient, and postoperative computed tomography showed no endoleak. This case report supports the feasibility of endovascular surgery for treating AAA in patients with a horseshoe kidney.

## Introduction

Open repair and endovascular aneurysm repair (EVAR) are two treatment options for abdominal aortic aneurysm (AAA). Although EVAR is less invasive than artificial blood vessel replacement, it does have disadvantages, including anatomical constraints and the inability to reconstruct the inferior mesenteric arteries within the therapeutic range. We encountered a case of an AAA in a patient with a horseshoe kidney and accessory renal arteries for which we performed EVAR. As this case was associated with renal dysfunction, an aortic cuff was used in combination with accessory renal artery occlusion to prevent type II endoleak, and attempts were made to reduce the amount of contrast medium used during surgery. The surgical outcome was good, with only a slight transitory deterioration in renal function.

## Case Report

A 78-year-old man was found to have an AAA with a maximum diameter of 50 mm based on the computed tomography (CT) performed during the preoperative examination for the resection of his gastric cancer. A contrast-enhanced CT scan confirmed the presence of an infrarenal AAA that extended to the terminal aorta and was associated with a horseshoe kidney. The patient had diabetes mellitus and hyperuricemia, and he had undergone chemotherapy for esophageal cancer. His laboratory data were as follows: creatinine (Cr) 1.31 mg/dL, blood urea nitrogen 20.0 mg/dL, and estimated glomerular filtration rate (eGFR) 41.4 mL/min/1.73 m^2^. Upper gastrointestinal endoscopy showed advanced gastric cancer. The patient provided written informed consent for the publication of his data.

**Figure 1:**
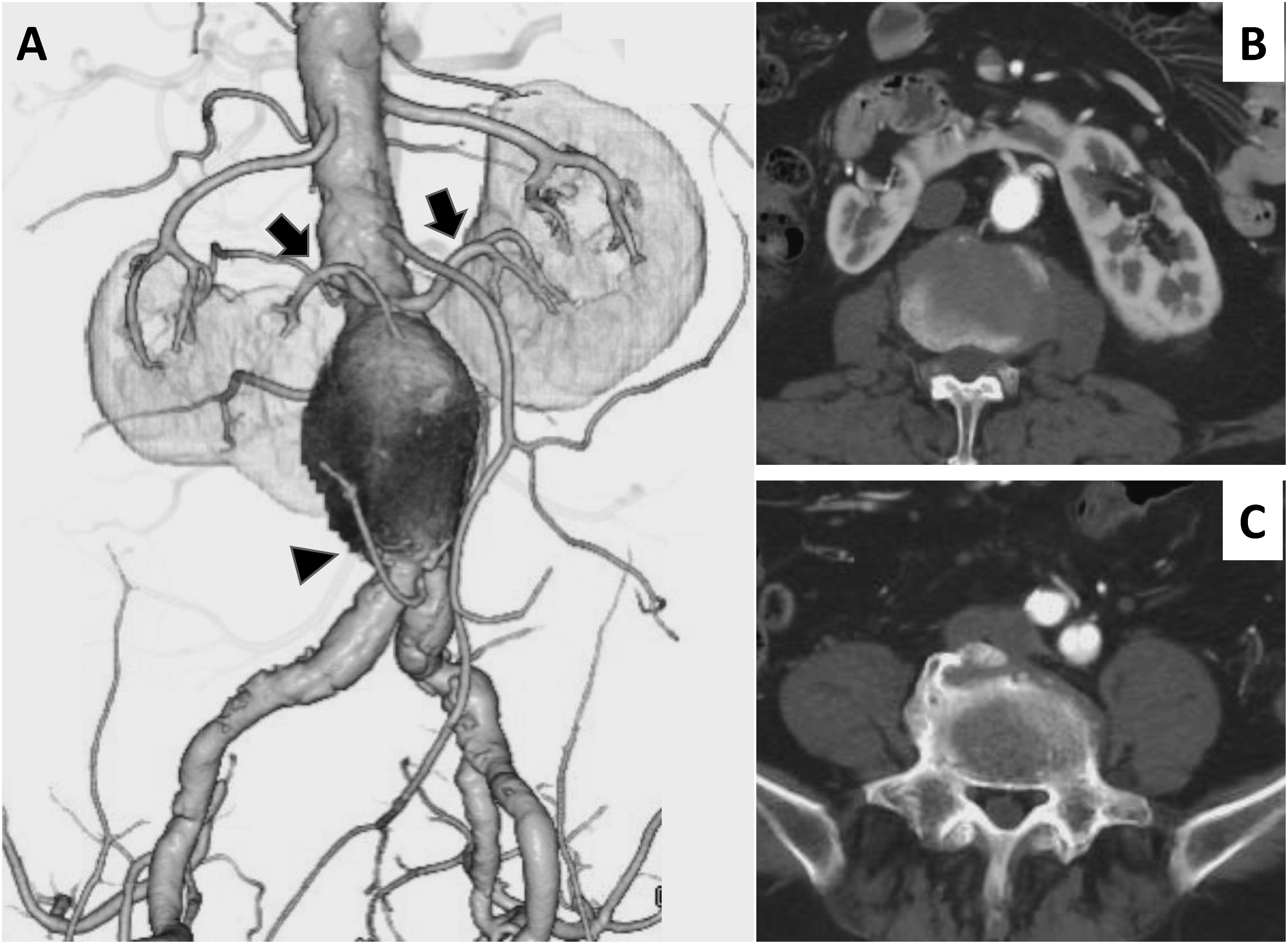
Fig. 1 Preoperative contrast-enhanced computed tomography (CT).

Contrast-enhanced CT showed an infrarenal AAA with a maximum diameter of 50 mm (**Fig. 1A**) and a horseshoe kidney with fusion of the inferior poles on the ventral side of the AAA. Three-dimensional CT revealed that the two main renal arteries and the inferior mesenteric artery (IMA) formed the non-aneurysmal proximal aortic neck. Furthermore, two accessory renal arteries originated from the proximal aneurysm sac (**Fig. 1B**). The diameters of the IMA and both accessory renal arteries were 4.0 and 3.9 mm, respectively. The diameter of the blood vessel from the IMA bifurcation to the ectopic renal artery bifurcation was approximately 20 mm. Another accessory renal artery, with a diameter of 2.5 mm, was observed from the right common iliac artery (CIA) to the renal isthmus (**Fig. 1C**). There were also seven lumbar arteries.

(**A**) Three-dimensional CT: 50-mm spindle-shaped aneurysm seen under the renal artery. The accessory renal artery has two branches (→) from the abdominal aorta and one branch (▶) from the left and right common iliac arteries. (**B**) Axial slice of contrast-enhanced CT: The accessory renal artery bifurcates from the abdominal aorta. (**C**) Axial slice of contrast-enhanced CT: Accessory renal artery from the left common iliac artery (→).

Considering that this patient was 78 years old and was scheduled to have open surgery for the treatment of his gastric cancer, the life prognosis of gastric cancer after surgery was expected to be more than 2 years; thus, we decided to treat this condition using EVAR. Occlusion of the two accessory renal arteries and the IMA was used to prevent type II endoleak; however, coil embolization of each of these resulted in a longer fluoroscopy time, increased radiation exposure, and an increased use of contrast media. In consideration of the patient’s preoperative renal dysfunction, the amount of contrast media used needed to be minimized. Hence, an aortic cuff was used to easily and reliably occlude the IMA and two accessory branches of the renal artery by placing an Excluder® (WL Gore and Associates, Flagstaff, AZ, USA) aorta extender.

**Figure 2:**
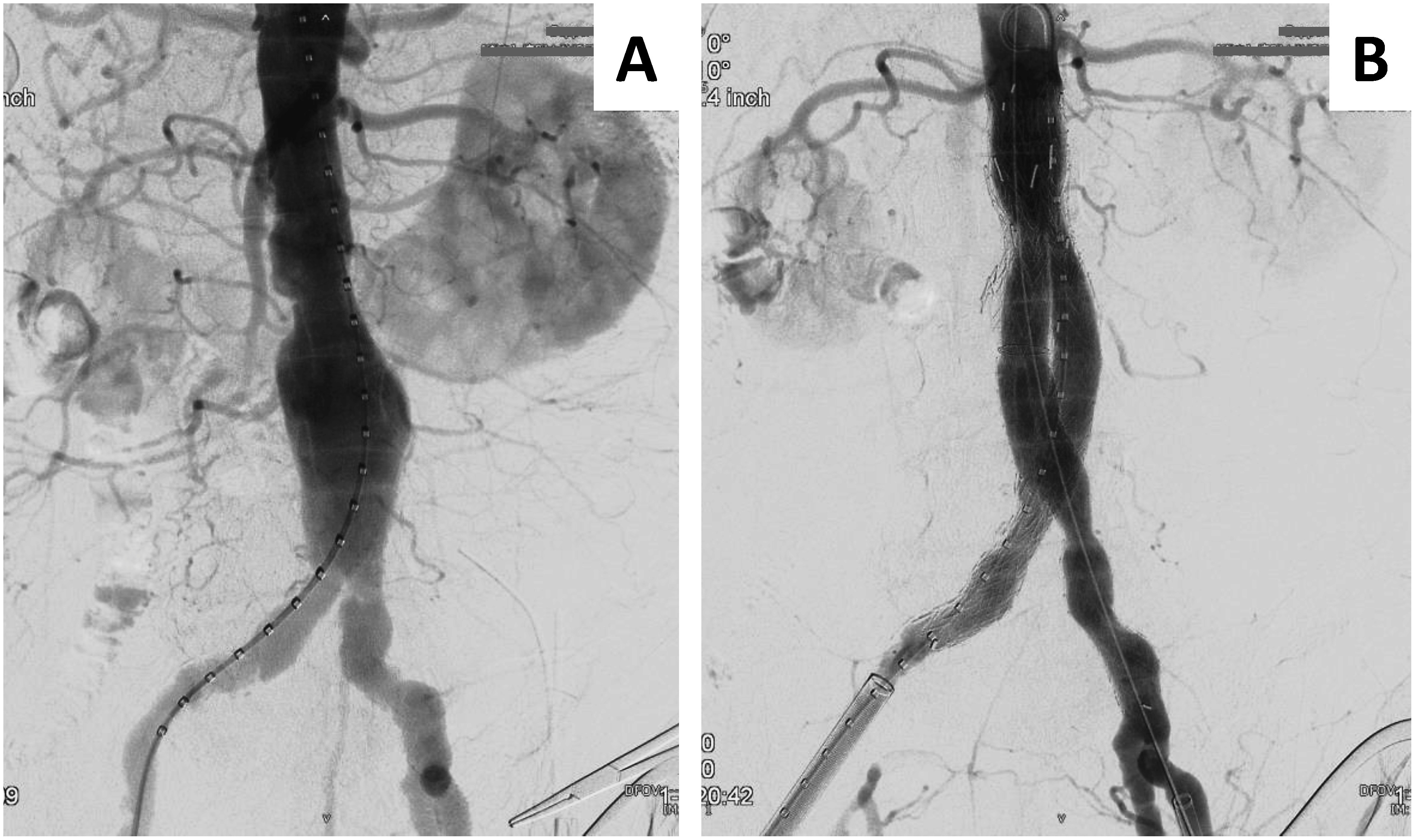
Fig. 2 Intraoperative imaging.

Surgery was performed under general anesthesia. The common femoral artery was exposed. The peripheral aorta was imaged from the level of the renal artery (**Fig. 2A**). First, the aorta extender (PLA320400J) was placed to occlude the origin of the accessory renal artery and IMA branching from the vicinity of the aneurysm center to prevent a type II endoleak. Since the accessory renal arteries arose from the vicinity of the upper edge of the aneurysm sac, a 32-mm cuff was used as a seal for the accessory renal artery erasure. Further, the Trunk-Ipsilateral Leg Endoprosthesis (RLT231416J) was deployed below the renal artery beyond the right CIA covering the third accessory renal artery. A contralateral iliac leg (PLC141400J) was deployed at the left CIA. Intraoperative imaging showed only a slight type II endoleak from the lumbar artery in the remaining aneurysm (**Fig. 2B**). The fluoroscopy and operative times were 17 and 87 min, respectively. Forty milliliters of contrast medium were used.

(**A**) Before stent graft deployment. (**B**) After stent graft deployment. Only a type II endoleak from the lumbar artery is seen. The aorta extender is placed, and type II endoleaks from the inferior mesenteric artery and accessory renal artery are not observed.

There were no major complications after the operation. Postoperative renal function temporarily worsened with a Cr of 1.88 mg/dL and eGFR of 27.9 mL/min/1.73 m^2^ on the third postoperative day; however, Cr and eGFR were 1.51 mg/dL and 35.4 mL/min/1.73 m^2^ on the tenth postoperative day. The patient’s renal function continued to improve, and no further deterioration was observed.

**Figure 3:**
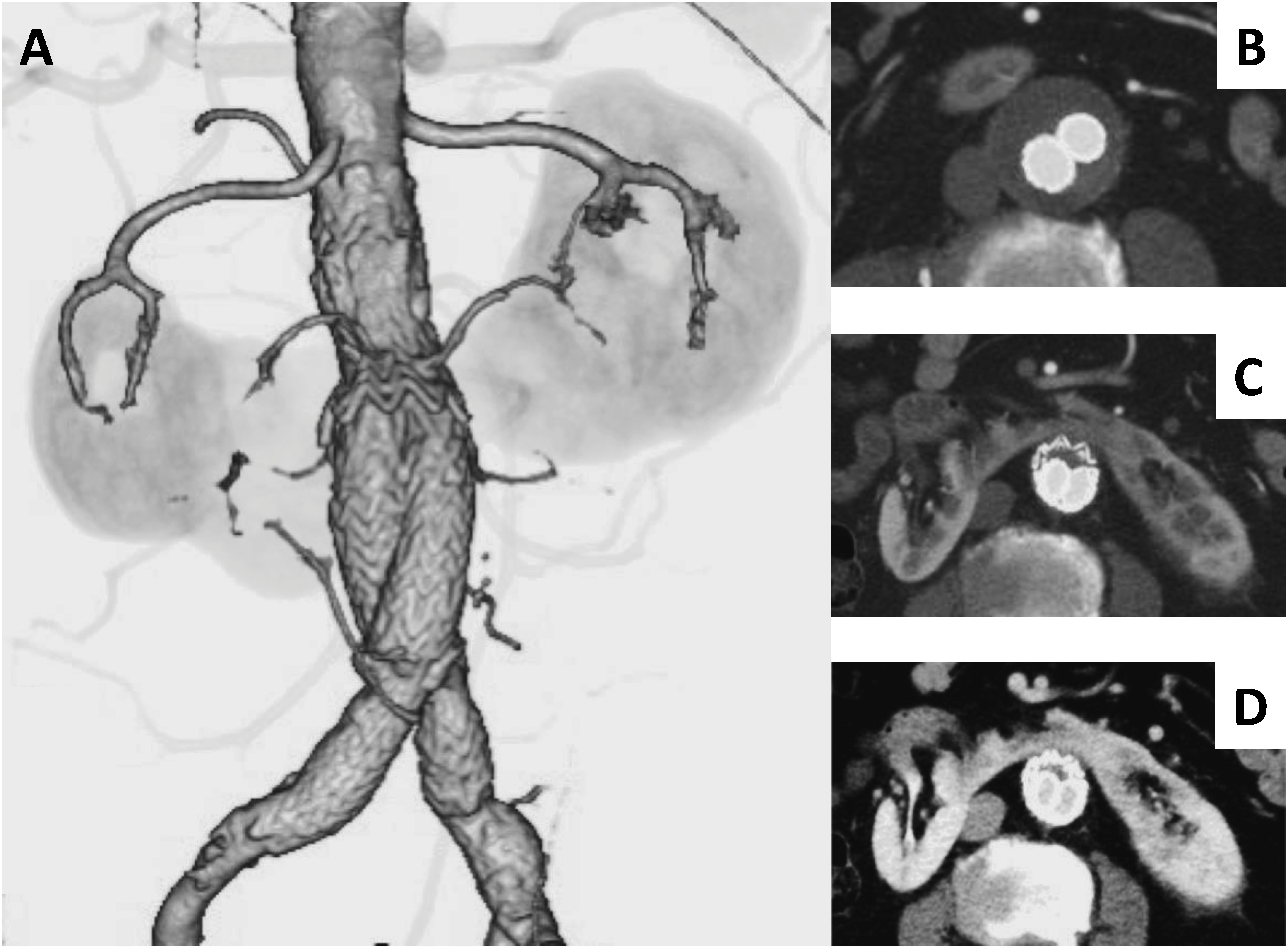
Fig. 3 Postoperative contrast-enhanced computed tomography (CT).

A postoperative contrast-enhanced CT scan showed that the IMA and two accessory renal arteries were obstructed by the aorta extender. The peripheral side of the accessory renal artery was imaged, but the collateral circulation was not identified (**Fig. 3A**). There was no endoleak and aneurysm thrombosis was obtained after surgery (**Fig. 3B**). In the early phase of contrast enhancement, the contrast effect in the renal isthmus was poor (**Fig. 3C**); however, in the delay phase (**Fig. 3D**), there was no clear renal infarction.

(**A**) Postoperative three-dimensional CT: Aorta Extender (➡) blocks the origin of the inferior mesenteric artery and the accessory renal artery; however, blood flow is confirmed in the accessory renal artery. (**B**) No endoleak is observed, and aneurysm thrombosis is obtained after surgery. (**C**) In the early phase, the contrast effect in the renal isthmus is poor. (➡) (**D**) In the delayed phase, the renal isthmus is imaged to the same extent. (➡).

## Discussion

Horseshoe kidney is a rare congenital anomaly characterized by the fusion of the lower poles of the kidneys, with an incidence of 0.25%.^1)^ Crawford and Crawford^2)^ reported that AAA was combined with a horseshoe kidney in only 6 out of 1,100 AAA surgical cases.

Open repair and EVAR are available for the treatment of AAA combined with a horseshoe kidney; however, indications for and selection criteria of these options are still controversial. Open repair has the advantage of reconstruction of the accessory renal arteries; however, there is a possibility of ureteral damage during reconstruction of the accessory renal arteries and isthmus owing to the abnormal course of the ureters. There are reports on the preservation of the renal isthmus without complications even when the renal isthmus was cut.^3)^ On the other hand, as there are concerns about complications such as hemorrhage, urinary extravasation, and infection after resection of the renal isthmus, this technique must be used with caution.^4)^ By selecting the retroperitoneal approach during open repair, it is possible to approach aneurysms even with the accessory renal artery or ureteral abnormalities. However, kidney injury may be caused by its dislocation.

In the case of our patient, the renal parenchyma in the renal isthmus was thick, and complications, such as bleeding owing to disconnection of the renal isthmus, were feared. Considering that the patient was undergoing treatment for esophageal cancer and required open gastric surgery for gastric cancer, we chose to perform EVAR.

In recent years, case reports on EVAR for the treatment of AAA combined with a horseshoe kidney have been published^5)^; however, problems with EVAR in these cases included a type II endoleak owing to the accessory renal arteries, renal infarction because of accessory renal artery occlusion, and renal dysfunction.

Regarding an endoleak owing to the accessory renal artery, a previous report^6)^ suggested that it is not necessary to add embolization for the prevention of postoperative endoleak because the artery is an end artery. However, another report showed rupture of the AAA owing to a type II endoleak from the accessory renal artery.^7)^ The reason for this is thought to be that the accessory renal artery, which is terminal artery, is slightly communicated from the main renal artery. In the present case, there were two accessory renal arteries from the vicinity of the upper edge of the aneurysm sac, as well as from the IMA, lumbar artery, and many branching arteries. It is common to embolize the origin of each artery with a coil. However, in this case, as multiple arteries needed to be embolized, there were concerns about disadvantages, such as a longer fluoroscopy time, increased radiation exposure, and increased use of contrast media. Hence, instead of embolizing each artery individually by placing an aortic cuff in the accessory renal artery bifurcation in advance, the origin of the artery was occluded.

Other challenges with EVAR for AAA combined with a horseshoe kidney with accessory renal arteries include postoperative renal infarction and renal dysfunction owing to the sacrifice of the accessory renal artery. Dorffner et al.^8)^ suggested blocking up to 32% of the total blood flow with a stent graft if renal function is normal. Güven et al.^9)^ reported no obvious complication even when the accessory renal artery with a diameter of 6 mm was occluded with a stent graft. Ruppert et al.^10)^ indicated the safety of EVAR using the Eisendrath classification as follows: if renal function is normal, the Eisendrath classification types I and II with one renal with one renal artery on each side are suitable for EVAR; EVAR should be performed carefully in types III and IV with two renal arteries on each side; and EVAR should not be performed in type V with three or more renal arteries on each side.

This case may be classified as type IV with two right and two left renal arteries and one accessory artery from the right CIA to the renal isthmus. The accessory renal arteries were thin at less than 4 mm in diameter, and preoperative renal dysfunction was mild; therefore, it was expected that there would be little effect on renal function after accessory renal artery occlusion, so treatment with EVAR was considered to be favorable. Contrast-enhanced CT after surgery confirmed blood flow to the accessory renal artery that blocked the origin, and postoperative renal dysfunction was transient. This suggests the development of collateral circulation to the accessory renal artery. Therefore, we consider it necessary to gain more experience with cases involving the formation of collateral circulation after occlusion of the accessory renal artery, both in the presence and absence of renal dysfunction, and to continue studying this tissue.

## Conclusion

Horseshoe kidney associated with AAA is a very rare condition. We performed EVAR using an aortic cuff in a patient diagnosed with AAA with a horseshoe kidney and coexisting accessory renal arteries, and satisfactory results were obtained without postoperative complications such as permanent renal dysfunction.
